# Neurophysiological Correlates of Gait in the Human Basal Ganglia and the PPN Region in Parkinson’s Disease

**DOI:** 10.3389/fnhum.2020.00194

**Published:** 2020-06-04

**Authors:** Rene Molina, Chris J. Hass, Kristen Sowalsky, Abigail C. Schmitt, Enrico Opri, Jaime A. Roper, Daniel Martinez-Ramirez, Christopher W. Hess, Kelly D. Foote, Michael S. Okun, Aysegul Gunduz

**Affiliations:** ^1^Department of Electrical and Computer Engineering, University of Florida, Gainesville, FL, United States; ^2^Norman Fixel Institute for Neurological Diseases and the Program for Movement Disorders and Neurorestoration, University of Florida, Gainesville, FL, United States; ^3^Department of Applied Physiology and Kinesiology, University of Florida, Gainesville, FL, United States; ^4^J. Crayton Pruitt Department of Biomedical Engineering, University of Florida, Gainesville, FL, United States; ^5^School of Kinesiology, Auburn University, Auburn, AL, United States; ^6^Tecnológico de Monterrey, Escuela de Medicina y Ciencias de la Salud, Monterrey, Mexico; ^7^Department of Neurology, University of Florida, Gainesville, FL, United States; ^8^Department of Neurosurgery, University of Florida, Gainesville, FL, United States

**Keywords:** Parkinson’s disease (PD), brainstem, deep brain stimulation (DBS), gait, DBS, deep brain stimulation

## Abstract

This study aimed to characterize the neurophysiological correlates of gait in the human pedunculopontine nucleus (PPN) region and the globus pallidus internus (GPi) in Parkinson’s disease (PD) cohort. Though much is known about the PPN region through animal studies, there are limited physiological recordings from ambulatory humans. The PPN has recently garnered interest as a potential deep brain stimulation (DBS) target for improving gait and freezing of gait (FoG) in PD. We used bidirectional neurostimulators to record from the human PPN region and GPi in a small cohort of severely affected PD subjects with FoG despite optimized dopaminergic medications. Five subjects, with confirmed on-dopaminergic medication FoG, were implanted with bilateral GPi and bilateral PPN region DBS electrodes. Electrophysiological recordings were obtained during various gait tasks for 5 months postoperatively in both the off- and on-medication conditions (obtained during the no stimulation condition). The results revealed suppression of low beta power in the GPi and a 1–8 Hz modulation in the PPN region which correlated with human gait. The PPN feature correlated with walking speed. GPi beta desynchronization and PPN low-frequency synchronization were observed as subjects progressed from rest to ambulatory tasks. Our findings add to our understanding of the neurophysiology underpinning gait and will likely contribute to the development of novel therapies for abnormal gait in PD.

**Clinical Trial Registration:** Clinicaltrials.gov identifier; NCT02318927.

## Introduction

The pedunculopontine nucleus (PPN) region has recently generated much interest as a deep brain stimulation (DBS) target for the treatment of freezing of gait (FoG) in Parkinson’s disease (PD; Stefani et al., 2007, 2013; Moreau et al., 2009; Moro et al., 2010; Khan et al., 2012; Welter et al., 2015; Hamani et al., 2016). Surgically targeting the PPN region has proven challenging due to the lack of clear anatomical demarcations on traditional imaging. Outcomes from small clinical trials have shown mixed benefits (Zrinzo et al., 2008, 2011; Mestre et al., 2016). A better electrophysiological understanding of the PPN region will be critical to the development of future therapeutic strategies and especially to closed-loop neuromodulation which relies on the identification of a control signal. Several clues point to the PPN region as an important node in human gait-related activities. First, it has been shown that gait-related symptoms have been closely associated with diseases associated with prominent PPN degeneration (e.g., PD and progressive supranuclear palsy). Second, the PPN has been shown in animals to be a critical node in the gait cycle. Finally, altered gait has been reported in lesional studies of the PPN region, and gait deficits are improved by electrical stimulation in both rat and non-human primate PD models (Aziz et al., 1998; Breit et al., 2001, 2005; Jenkinson et al., 2006; Nandi et al., 2008; Karachi et al., 2010; Grabli et al., 2013).

The globus pallidus internus (GPi) and the subthalamic nucleus (STN) are common targets for DBS therapy and have been utilized to address dopamine responsive symptoms in PD (e.g., tremor, rigidity, bradykinesia), as well as medication-related motor fluctuations and dyskinesia. However, both targets have failed to adequately address gait-related issues (Heremans et al., 2013; Morita et al., 2014). One approach to address this important gap in treatment has been to augment GPi or STN DBS with PPN region DBS (Stefani et al., 2007; Moreau et al., 2009).

The current study aimed to record simultaneously from the human PPN region and the GPi to elucidate the electrophysiological correlates of human gait. The study was part of a Michael J. Fox Foundation challenge grant to improve neuromodulation approaches in patients with gait and freezing issues not responsive to dopamine replacement therapies. Also, patients included in the study were not required to possess a 30% improvement with dopaminergic medication. The data presented here is collected from electrodes implanted bilaterally in the PPN region and bilaterally in the posteroventral GPi from five ambulatory human participants with PD. Local field potentials (LFPs) were collected chronically during the no active stimulation condition through bidirectional DBS implants.

## Materials and Methods

### Subjects

University of Florida Institutional Review Board and FDA IDE (G140181) approvals were granted for the recruitment of five human PD subjects (two females; age range: 50–74 years). A confirmed medical history of on and off dopaminergic medication FoG was required, despite exhaustive dosage optimization by a movement-disorder trained neurologist. Additionally, participation required more than two FoG episodes per month, a score of more than 1 on item #3 on the Freezing of Gait Questionnaire (FOG-Q; Giladi et al., 2000), and also a minimum of five FoG episodes under a provocation gait protocol. Study candidates were screened and evaluated at the Norman Fixel Institute for Neurological Diseases at the University of Florida by an interdisciplinary clinical-research DBS team. The public listing with the full inclusion and exclusion criteria can be found on ClinicalTrials.gov (NCT02548897). Seven potential candidates were screened, from which five subjects (aged 50–75, two females) were recruited and provided written informed consent before any study interventions (Table 1). Subject 3 deteriorated very quickly after surgery and could only complete tasks by utilizing a walker.

**Table 1:** Subject demographics.

Subject ID	Years Since	L-DOPA	L-DOPA	Mean UPDRS III		Mean UPDRS IV	
	PD Diagnosis	OFF Freezer	ON Freezer	(Screening/6 Month)		(Screening/6 Month)	
1	9	Yes	Yes	26	22	5	7
2	10	Yes	Yes	23	17	5	3
3	23	Yes	Yes	30	40	5	6
4	9	Yes	Yes	24	21	14	5
5	5	Yes	Yes	35	33	5	6

*Legend: L—dopa-levodopa, UPDRS—Unified Parkinson’s Disease Rating Scale, UPDRS III—motor and IV-fluctuations in therapy*.

### Device and Implantation Surgery

The implantation procedure was divided into three stages. Two leads were unilaterally implanted in the first stage, and in the second stage of the operation 2 to 4 weeks later the other two leads were implanted contralaterally. In one subject the two PPN leads were placed in one stage and the two GPi leads in a second stage which was performed 2 to 4 weeks later. The final stage of the operation occurred approximately 4 weeks after stages one and two and in this stage, the four DBS leads were connected to the implantable neurostimulators (INS) and secured in a sub-clavicular pocket. Each of the three stages was separated by a month to resolve brain edema, minimize brain shift, and to avoid prolonged time in the operating room. Each subject received two quadripolar Medtronic model 3,387 leads targeting the GPi, bilaterally, and two model-3,389 leads targeting the PPN, also bilaterally. Both GPi leads were connected to a Medtronic Activa PC+S (Medtronic PLC, Minneapolis, MN, USA) in a sub-clavicular pocket on the left side, and both PPN leads were connected to another Activa PC+S in a different sub-clavicular pocket on the right. Pairing the leads by nuclei instead of laterality was performed to facilitate the preferred differences in therapeutic frequencies for each brain target. Traditionally, the PPN has been stimulated at lower frequencies than the GPi and a single IPG cannot drive two leads with differing frequencies. Nuclei targeting was achieved through high-resolution MRI imaging performed before surgery. A stereotactic head CT was coregistered to T1 and FGATIR MRI images. Additionally, these images were fused to a patient-specific, 3-D deformable brain atlas (Sudhyadhom et al., 2012). During the implantation surgery, the subjects remained awake for microelectrode recordings performed using an FHC 4000 LP+ system (FHC, Bowdoin, ME, USA). A movement-disorder neurologist evaluated changes in neural firing derived from the microelectrode recordings to refine the physiological mapping of the nuclei borders of each target. Since the PPN region has not been shown to exhibit clear demarcations on MRI images and has not been demonstrated to have widely accepted and reproducible physiological markers during MER mapping, the PPN region targeting relied more on interpolation from surrounding structures and measurements than from 4th ventricular landmarks (Hamani et al., 2007; Zrinzo et al., 2008; Shimamoto et al., 2010; Morita et al., 2014). Post-operative CT images co-registered with pre-operative MRI was used to confirm the post-operative position of the leads.

### Postoperative Data

Monthly electrophysiological data collections commenced approximately 30 days after the last phase of the surgical procedures and were performed acutely in a gait laboratory equipped with a 10-camera motion capture system (Vicon Motion Systems, Oxford, UK) and three embedded force plates (Bertec, Newton, MA, USA). The Activa PC+S recorded from two electrodes in a symmetric bipolar configuration for common-mode rejection. The bipolar contacts were separated by a middle contact, which could be used for stimulation in a monopolar configuration (Afshar et al., 2012; Stanslaski et al., 2012; Bourget et al., 2015). Although the data presented here were collected in the absence of stimulation, we aimed to identify gait features that could guide in responsive stimulation. Neural data were sampled at 421.9 Hz. Concurrent EMG and inertial (accelerometer, gyroscope, and magnetometer) data were recorded with wireless sensors (Delsys, Inc., Natick, MA, USA) sampled at 1,925.93 Hz and 148.25 Hz, respectively. To synchronize the neural signals with the external sensor data, the PC+S delivered a low-frequency marker burst (~5 Hz in the GPi and ~8 Hz in the PPN). A nearby EMG sensor (on the neck or forehead) was able to detect the onset and termination of the low-frequency marker bursts. The data were also supplemented with 24 frames/s of video recordings. Short synchronization pulses were ported to the EMG amplifier that simultaneously flashed a LED captured by the video camera. Each modality was linked through marker bursts or EMG/LED pulses and the data were conjoined manually in MATLAB. The protocol was comprised of a battery of tasks, performed both on and off dopaminergic medication, to study gait and to elicit FoG, including stepping in place, changing cadence, narrow passages, dual-attention tasks, turning, and navigating obstacles (Snijders et al., 2008, 2010, 2012; Spildooren et al., 2010; Stegemõller et al., 2012). Subjects restricted dopaminergic medication for at least 12 h before the off medication data collections. Recurrent data collections over 5 months post-implant were conducted during the patients’ monthly check-up/programming visits.

### Signal Processing and Statistics

Signal processing was performed within MATLAB 2018a (MathWorks, Natick, MA, USA) with custom scripts. Power spectral densities (PSD) were calculated using a 40-order autoregressive model (Schalk et al., 2004; Stoica and Moses, 2004). The data was windowed at 250 ms and with a 50% overlap. Each spectral bin within a condition was tested for significance *via* the Mann–Whitney test with Bonferroni correction. Mean power distributions between two conditions were tested for significance with the Kruskal–Wallis test with Dunn’s corrected test for multiple comparisons. Multiple linear regressions were done to compare the PPN feature band against gait measures for all subjects. Individual regressions were done for conditions and variables that reached significance at the group level. *F*-tests were used to test the regressions for significance. Spectral bins that were significant were labeled as “feature detection.” We picked the channels with the strongest feature during the “feature extraction” step. This approach is commonly used by our laboratory and other laboratories performing similar experiments. In choosing contacts for analysis, we chose a method to avoid picking a single combination *a priori*, not knowing where the difference in modulation would be found. Since multiple contact combinations and hemispheres showed a difference, for subsequent analysis, the combination that showed the largest difference was used.

### Clinical Scores and Assessment

Each monthly visit included biomechanical gait measures (e.g., gait speed, cadence, stride length) collected *via* the Vicon motion capture system and processed by the Applied Neuromechanics Laboratory at the University of Florida. The subjects were administered the FoG-Q, the Gait and Falls Questionnaire (GFQ; Giladi et al., 2000), the Activities/Balance Confidence Scale (ABC; Powell and Myers, 1995), the PD Quality of Life Questionnaire (PDQ; Peto et al., 1998), and the Unified Parkinson’s Disease Rating Scale (UPDRS; Fahn and Elton, 1987). Assistive-walking-device information was also collected. These measures were assessed during screening and at baseline (before DBS implantation). Clinical scores and assessments were primarily used to evaluate the effectiveness of responsive PPN DBS and to track disease progression and the clinical outcomes (Molina et al., 2018). An independent movement disorders neurologist reviewed the videos recorded during data collections. The neurologist labeled freezing episodes, the type of freezing (such as during walking, start hesitation, or turning hesitation), the severity of the freeze, and the confidence in the label of a freezing event.

## Results

**Figure 1:**
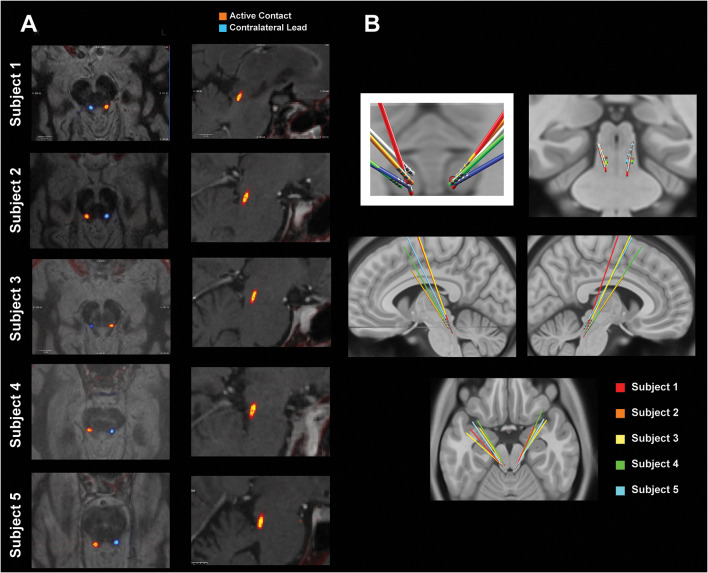
Pedunculopontine nucleus (PPN) lead localization methods. **(A)** MRI-CT co-registered images of each subject. The “active contact” marks the center of the bipolar electrode configuration that yielded the feature most correlated with gait. The left panels show T1 inverted axial views of the active contact (orange) and the contralateral lead (blue). The right panels show T2 sagittal views of the active contact. **(B)** Leads normalized to MNI space using Lead-deep brain stimulation (DBS). All leads are shown with a red dot that roughly defines the location of the PPN.

**Figure 2:**
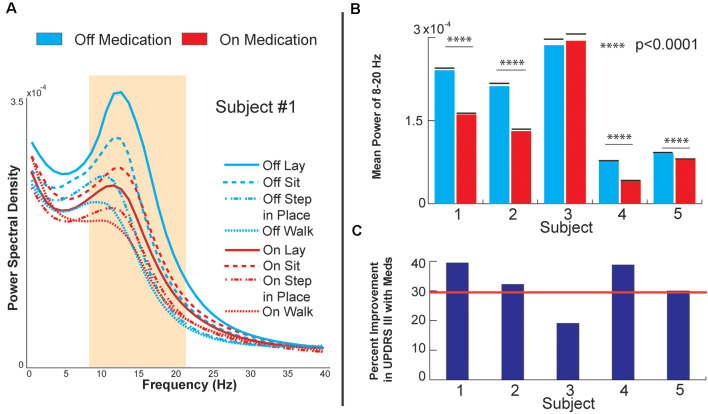
Globus pallidus internus (GPi) response to medication. **(A)** Power spectral density of GPi signals from Subject 1, OFF, and ON levodopa medication during various baseline and gait tasks. **(B)** Bar plots of raw decrease in GPi beta power during sitting (8–20 Hz). **(C)** Bar plots of each subject’s percent improvement in UPDRS III score from OFF medication to ON medication during sitting. The red line marks a 30% improvement, a commonly employed clinical benchmark for medication responsiveness.

**Figure 3:**
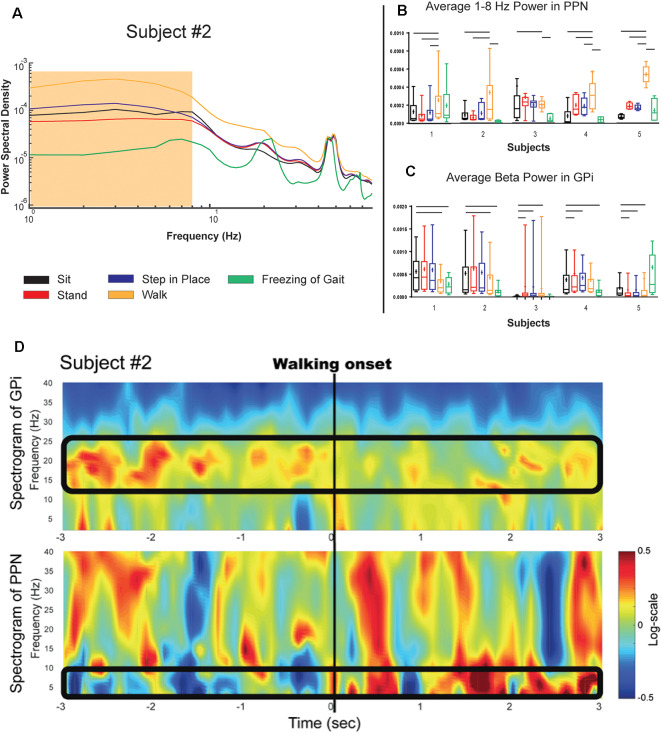
PPN and GPi response to gait tasks in the on medication condition. **(A)** Logarithmic power spectral density of PPN signal power in a single subject during various balance and gait-related tasks. **(B,C)** Single-subject (log normalized to standing) demonstrating PPN low frequency and GPi beta modulation with gait onset shown as boxplots. Significance bars indicate that tasks that statistically could be differentiated from gait every month (*p* < 0.001). Panel **(D)** Shows the temporal evolution of neural signatures with gait initiation and the spectrogram from the same subject as **(A)** averaged across gait initiation trials in one session. This subject exhibited a beta peak while at rest, and this beta peak decreased with gait initiation, while the PPN modulated in response to walking in the 1–8 Hz spectral band. The outlined bands show the dissipation of the beta rhythm in the GPi while the PPN feature bands modulated at the onset of gait.

Due to the variation in PPN localization, two methods were used to assess the location of the recorded electrophysiology. Figure 1A shows T1 inverted (left panels) and T2 (right panels) MRI images of the active contact (middle) from the symmetric bipolar recordings shown in Figures 2, 3. The rostral face of the PPN region resides in an area laterally and ventrally bound by the spinothalamic tract, the lateral lemniscus, and medial lemniscus, as well as dorsal-medially by the spinal cerebellar peduncle (SCP) decussation (Hamani et al., 2016). Visual inspection of imaging, shows that subjects 1–4 were generally within this area, while subject 5’s leads were dorsal to the region. Additionally, anatomically the rostral part of the PPN region is in the plane of the mid-inferior colliculus. T2 images confirmed the active DBS contact to be at or below this plane for all five subjects. Figure 1A reveals the active contacts in the PPN region. Figure 1B shows a second confirmatory method that was used to determine DBS lead placement, using Lead-DBS to normalize the individual images to the MNI brain space. Collectively by both methods, the leads are shown to be in an estimated PPN region (Snijders et al., 2016). The leads show a consistent cluster. The additional analysis did not reveal a relationship between the spatial location of the active contact and the corresponding clinical score. The locations marked as “active contact” in Figure 1A depict the contact from which stimulation was provided for therapy. These contacts were chosen for therapy as the bipolar recordings from their two adjacent electrodes yielded the highest PPN neural correlate for gait in the absence of stimulation.

Figure 2A shows the PSD of GPi signals in one of the subjects tested under two different medication conditions while performing different tasks. Each task was performed for 3 min and aggregated across five consecutive months. In all the conditions, a peak in the low beta range (8–22 Hz) in at least one bipolar combination and one hemisphere is prominent. This beta peak modulates in response to the task and the medication state, and specifically, the peak decreases with medication intake (red vs. blue lines in Figure 2A). Figure 2B shows the mean power in the low beta frequency range for each subject. A decrease in low beta was observed in response to dopaminergic medication intake across four of the five subjects, with Subject 3 lacking a decrease in low beta. Baseline pre-operative clinical improvement response to medication was measured as a percent reduction in UPDRS III PD motor score off and then on medication in this study cohort. Figure 2C shows the percent improvement in UPDRS III scores in response to the medication at the pre-operative baseline. Except for Subject 3, the cohort met the usual clinical benchmark of a 30% improvement off to on dopaminergic medications. Subject 3 did not exhibit either medication-induced beta suppression in GPi or minimal improvement (<30%) in clinical scores with medication. Figure 3 summarizes the spectral modulation in the GPi and the PPN during different walking tasks during the on medication condition. A full dataset of walking tasks during off medication per the protocol was not available and is therefore not shown. Each task was performed for 3 min and aggregated across five consecutive months. The power spectral density of a single subject is shown in Figure 3A. There was an increased PPN activity in the sub-8 Hz spectral range with axial and gait trials compared to sitting and standing. The data reveal differences in walking and rest for all subjects and differences between most other tasks. As subjects progressed through sitting, standing, stepping-in-place, and walking, the PPN 1–8 Hz feature modulated in response to the task, reaching maximum power during walking in four out of five patients. The significance bars denote tasks that were differentiable from gait across all recordings within 6 months with *p* < 0.01. The walking task was the most distinguishable from the other tasks in all subjects except for Subject 3, who had to perform the stepping-in-place and walking tasks using a walker due to rapid disease progression. The stimulation contact was chosen the be in the center of the two electrodes that yielded the most significant PPN 1–8 Hz feature. This feature was observed to be closer to the rostral part of the PPN region which was located near the level of the mid-inferior colliculus (see Figure 1A). Figure 3B (PPN) and Figure 3C (GPi) are boxplots of the features of interest. Figure 3B shows a decrease in the 1–8 Hz band with freezing of gait, albeit there were few episodes captured in the laboratory (3–12 episodes). Figure 3C summarizes the data from GPi and it reveals a decrease in beta power from rest to gait initiation. Figure 3D shows the temporal evolution of neural signatures with gait initiation and the spectrogram from the same subject as Figure 3A averaged across gait initiation trials in one session. This subject exhibited a beta peak while at rest, and this beta peak decreased with gait initiation, while the PPN modulated in response to walking in the 1–8 Hz spectral band. The outlined bands show the dissipation of the beta rhythm in the GPi while the PPN feature bands modulated at the onset of gait.

**Figure 4:**
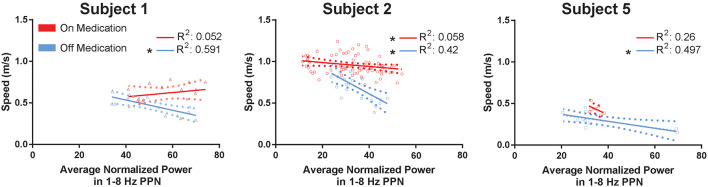
PPN low-frequency feature compared against gait metrics. The mean walking speed of subjects 1, 2, and 5 are plotted against their averaged normalized power in the 1–8 Hz PPN feature band. Each regression is performed by subject and within the same medication condition, for a total of six regressions. Each point represents a trial. Error bars are shown as dotted parabolas following the trend line. *p < 0.1.

The 1–8 Hz feature band was tested against measured cadence, walking speed, and stride length in multiple linear regression, where power in the feature band was the dependent variable and cadence, walking speed, and stride length were the predictors. Four different multiple regressions were done, during off and on medications, and testing both the lead that yielded the most reliable modulation between conditions in Figure 3 and the contralateral lead. The regressions using power from the reported lead reached significance (*p*-value 0.00027 and 0.016, off and on medication, respectively), while the regressions for the contralateral lead did not (0.066 and 0.15, off and on medication, respectively). The only feature to produce significance in the regressions in the available subjects was the PPN 1–8 Hz feature against walking speed (0.0076 and 0.0019, off and on medication, respectively). The individual regressions are shown in Figure 4. Complete gait metrics were available for three subjects (subjects 1, 2, and 5). Recording conditions or missing alignment artifacts precluded the inclusion of data from the other two subjects. Additionally, the location of the correlated feature was not the same bipolar contact for Subject 2 as shown in Figure 1, but it was observed on the same (ipsilateral) lead. Finally, changes in medication state were observed. The OFF-medication state resulted in higher *r*^2^ values (0.591, 0.42, and 0.497, respectively) than the ON-mediation state (0.052, 0.058, and 0.26, respectively). The slopes were significant for all of the OFF-medication regressions, and only for the ON-medication data for subject 2.

## Discussion

Herein we report human electrophysiological recordings from the GPi and the PPN regions during various gait tasks in five subjects. Our findings showed suppression of low beta power in the GPi in response to medication, and a 1–8 Hz modulation in four subjects in the PPN region correlating with human gait in the on medication state. Significant decreases in the GPi beta band with medication corresponded to marked improvements in clinical scores, which increased our confidence in the signal quality captured with the bidirectional neurostimulators. GPi beta also decreased during gait tasks. Meanwhile, the 1–8 Hz power in the PPN region increased with walking and seemed to correlate with walking speed, which was not the case for GPi beta activity. GPi beta desynchronization and PPN activation in 1–8 Hz was shown as subjects progressed from rest to ambulatory tasks. Finally, postoperative imaging and normalization tools suggested that the PPN region was reasonably targeted and the DBS leads were well clustered, although the precise targets for DBS in this region remain unknown. Additionally, the small size of the cohort and the clustering of the active contact in the region may have prevented a clear delineation in the contact location by the subjects’ clinical response to the therapy (Goetz et al., 2019). The results from this study complement the current literature regarding low-frequency oscillations in the caudal region of the PPN and the relationship to human gait (Thevathasan et al., 2012).

The data further revealed a reproducible mean power change in the 1–8 Hz power band in response to gait initiation. Recent studies have reported a similar feature of low-frequency bands of interest in the PPN (Androulidakis et al., 2008; Thevathasan et al., 2012). However the data presented herein, collected over several months, did not reveal a distinct peak but rather changes in broad activity in the 1–8 Hz band. Variations in lead localization, the recording devices, and the experimental setup are a few of the many possible reasons for the difference between the feature reported herein and in other studies. Our group acknowledges that there is commonly uncertainty in the exact source of each LFP in human studies such as ours. This feature was observed to be closer to the rostral part of the PPN region which was located near the level of the mid-inferior colliculus. This 1–8 Hz power band will likely be important for guiding future studies and therapeutics.

An important observation from this study was the change in the correlation between walking speed and PPN activity in response to dopaminergic medication. Rodent and primate studies have suggested changes in neural activity in the on and off dopaminergic parkinsonian state (Mitchell et al., 1989; Futami et al., 1995; Breit et al., 2001, 2005; Matsumura and Kojima, 2002). It is reasonable that in the PD cases there is an important effect of medications and gait changes on efferent nuclei within the basal ganglia and brainstem. The current circuitry model broadly and nonspecifically suggests that gait is regulated by a large corticolimbic-ventral striatal-ventral pallidal-PPN-pontomedullary reticular nuclei-spinal cord network (Nutt et al., 2011). This network likely includes tonic inhibition of the mesencephalic locomotor region (MLR) through the GPi and the STN (Pahapill and Lozano, 2000; Hamani et al., 2007; Grillner et al., 2008; Alam et al., 2011). Figure 4 shows an example of GPi beta that is present during rest and dissipates at gait onset. Our data suggest the possibility that efferent brainstem nuclei are modulated with beta desynchronization in the GPi. It was interesting that the 1–8 Hz activity appeared during walking. The curious observation was the decrease in 1–8 Hz activity as the walking speed increased which conflicts with some of the published literature. Since this was only a five patient study and the study was of “on freezers” we would need to interpret this finding cautiously and we could not reliably conclude that 1–8 Hz power reflects an impairment of fast walking. The data also suggest that the PPN region has a role not limited to gait but is likely involved in tasks requiring balance and lower limb activation (i.e., standing and stepping-in-place).

Finally, we report GPi beta suppression with dopaminergic medication and a correlation to improvement in pre-operative clinical on-off medication scores. Data from Subject 3 was insightful and demonstrated that GPi beta suppression was not a direct consequence of levodopa intake but was more likely an indicator of symptomatic improvement as reflected in UPDRS scores. Beta power in multiple basal ganglia regions has been previously shown to correlate with symptom severity and its suppression, either through dopaminergic therapy or DBS and has been correlated with improvements in UPDRS scores (specifically bradykinesia, and rigidity; Brown et al., 2001; Levy et al., 2002; Kühn et al., 2006, 2009; Ray et al., 2008; Jenkinson and Brown, 2011). Excessive inhibitory (GABA-ergic) activity from the basal ganglia may dampen the activity of efferent nuclei such as in the brainstem PPN region. Furthermore, one may posit that a successful therapy may reestablish a healthier basal ganglia modulation, which in turn reduces rigidity and bradykinesia. A possible hypothesis for the lack of beta suppression in Subject 3 may be that under severe degeneration and lack of clinical response to dopamine, basal ganglia function cannot be restored, and beta power remains unchanged. This point will need further clarification as therapeutic strategies that target beta may be affected by disease progression.

Understanding the neural correlates of gait is crucial in defining future therapeutic strategies. The response to the beta band could dictate optimal dosage, administration times, or serve as a measure of disease progression. The roles GPi and the PPNregion each play in gait could also be harnessed to develop better therapies for walking disturbances.

We would also like to address potential artifacts in the dataset. The manufacturer, Medtronic, and their team of engineers recognize movements as a possible source of artifacts however these artifacts would produce large amplitude, broad bandwidth, narrow duration (concentrated) impulse responses in the spectrograms. Although Figure 3 does have broadband activity, this does not fit the narrow duration of movement artifact. Each subject had two devices, one for GPi leads and one for PPN region leads. If the PC+S was susceptible to movement artifacts presenting in the low-frequency band, then it would likely have shown up in at least one of the GPi recordings. None of the GPi recordings revealed a significant difference in the 1–8 Hz as was observed in the PPN data.

There were several important limitations of this study. This population suffered from debilitating gait and postural instability, from levodopa refractory FoG, from lower limb orthopedic issues, and other diseases, unrelated to PD or DBS. Obtaining high-quality recordings in different conditions was challenging in this population. Performing a large number of unassisted continuous walking trials was non-trivial. Subjects performed walking in short trials of ~8 m, followed by a spotter walking alongside the subject, and occasionally walking with an assistive device. Although recording conditions were not ideal, this study was able to collect neural data for the first time from ambulatory humans with a real-time recording from GPi and PPN regions. Furthermore, the few FoG episodes captured in only a few conditions and the required electrode configurations for recording also may have impacted the analysis. Second, we acknowledge the small sample size, although we would also argue that a small cohort facilitated greater depth of interaction with the subjects and also generated robust physiology datasets for analysis and interpretation. Moreover, this project was an early feasibility study of an invasive high-risk multi-lead procedure. We acknowledge that atypical parkinsonism could not be completely ruled out in these patients. However, without pathology or additional evidence of another disease, the clinical diagnosis for the cohort was idiopathic PD with “on” medication freezing. Finally, the localization of the human PPN region continues to be a formidable challenge. We presented individual data on localization using two methods and we purposely refer to this as a “region” rather than as a clearly demarcated target. However, the challenges presented in this study are not atypical and have been observed in the limited number of PPN DBS studies in the literature (Thevathasan et al., 2018).

To summarize, this study revealed the neural correlates of gait in human GPi and the PPN region in a population of patients with “on” dopaminergic gait and freezing issues. Real-time human electrophysiology revealed suppression of low beta power in the GPi in response to medication and gait tasks and a 1–8 Hz modulation in the PPN region that was correlated to ambulation.

## Data Availability Statement

The datasets from this study may be available upon request from the corresponding author with appropriate regulatory agreements.

## Ethics Statement

The studies involving human participants were reviewed and approved by US Food and Drug Administration (FDA) IDE G140181 and University of Florida (UF) IRB-01 201400951. The patients/participants provided their written informed consent to participate in this study.

## Author Contributions

RM collected data, analyzed data, and wrote the first draft of manuscript. CJH designed research, collected data, and edited manuscript. KS collected data, analyzed data, and edited the manuscript. AS, EO, and JR collected data and edited the manuscript. DM-R analyzed data and the manuscript. CWH edited the manuscript. KF performed surgeries. MO designed research and edited the manuscript. AG designed research, supervised research, and edited the manuscript.

## Conflict of Interest

The authors declare that the research was conducted in the absence of any commercial or financial relationships that could be construed as a potential conflict of interest.
